# Optimizing Scaled up Production and Purification of Recombinant Hydrophobin HFBI in *Pichia pastoris*

**DOI:** 10.3390/microorganisms13081845

**Published:** 2025-08-07

**Authors:** Mason A. Kinkeade, Aurora L. Pagan, Bryan W. Berger

**Affiliations:** 1 Department of Chemical Engineering, University of Virginia, Charlottesville, VA 22903, USA; qxa3tg@virginia.edu; 2 Germanna Community College, Fredericksburg, VA 22408, USA; den5tq@virginia.edu

**Keywords:** biosurfactant, hydrophobin, *Pichia pastoris*, HFBI, immobilized metal affinity chromatography, size exclusion chromatography, Capto MMC ImpRes, multimodal chromatography

## Abstract

Hydrophobins are small, surface-active protein biosurfactants secreted by filamentous fungi with potential applications in industries such as pharmaceuticals, sanitation, and biomaterials. Additionally, hydrophobins are known to stabilize enzymatic processing of biomass for improved catalytic efficiency. In this study, *Pichia pastoris* was used to recombinantly express hydrophobin HFBI from *Trichoderma reesei*, a well-characterized fungal system used industrially for bioethanol production. Iterative optimization was performed on both the induction and purification of HFBI, ultimately producing yields of 86.6 mg/L HFBI and elution concentrations of 48 μM HFBI determined pure by SDS-PAGE, over a five-day methanol-fed batch shake flask induction regiment followed by a single unit operation multimodal cation exchange purification. This final purified material represents an improvement over prior approaches to enable a wider range of potential applications for biosurfactants.

## 1. Introduction

**Figure 1:**
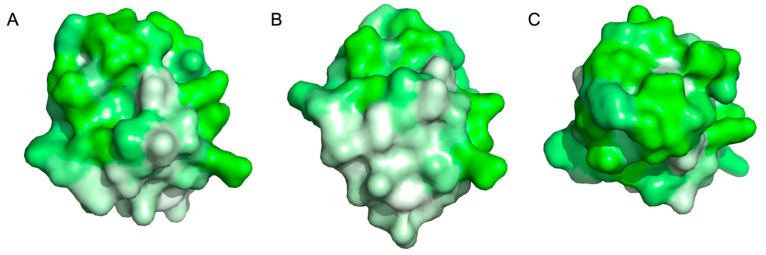
Hydrophobin HFBI hydrophobicity surface map with hydrophilic (green) and hydrophobic (white) regions highlighted: (**A**) side view showing amphiphilic tertiary structure; (**B**) bottom view showing hydrophobic patch; and (**C**) top view showing hydrophilic patch. Figure created in PyMOL v.3.1.6.1 using coordinates from protein data bank entry 2FZ6 and amino acid hydrophobicity classifications determined by Eisenberg et al. [9,10,11].

Biosurfactants are commonly found in nature, particularly in ecological decomposers such as filamentous fungi. These organisms secrete biosurfactants called hydrophobins, a class of small (<20 kDa) amphiphilic proteins, when breaking down biomass [1]. Hydrophobins are known to improve cellulose hydrolysis by facilitating fungal attachment to biomass and improving processive enzyme retention along cellulose fibers [2,3]. *Trichoderma reesei*, a filamentous fungus well known as an industrial workhorse for bioethanol production, co-secretes hydrophobin HFBI (Figure 1) alongside a cellulase, Cel7A, during biomass turnover [4,5]. This hydrophobin HFBI was chosen as the focus of this study due to the well-established body of knowledge on *T. reesei* [6,7,8].

Hydrophobins have applications in many different industries, often as a substitute for non-biological surfactants [12]. In pharmaceuticals, hydrophobins can be utilized as crystallization inhibitors to facilitate drug delivery at supersaturated concentrations [13]. Hydrophobin fusion proteins have been synthesized for applications such as antimicrobial coatings, plastic degradation, and cancer diagnostics [14,15,16]. Immobilized enzyme biosensors have been created without fusion proteins by submerging a hydrophobic surface in hydrophobin solution, allowing time for hydrophobin to self-assemble onto the surface, and then moving the now-modified surface into an enzyme solution for hydrophilic self-assembly [17].

Multiple methods of producing different hydrophobins can be found in the literature. *T. reesei* naturally secretes hydrophobins HFBI and HFBII when breaking down cellulose, but studies have shown the amounts of biosurfactant secreted are characteristic of ambient growth conditions rather than overproduction and use in industrial processes [4,18]. Attempts to produce hydrophobin HFBII in wild type *T. reesei* have generated low but scalable yields, peaking around 50 mg/L culture through employment of a specially designed fungal biofilm reactor [18]. Genetic engineering of *T. reesei* for HFBI overproduction, by adding two copies of the HFBI gene, drastically increased overall HFBI production to titers of 600–700 mg/L culture [19,20]. Over 80% of the HFBI produced, however, was trapped in *T. reesei* mycelium, causing low recovery of 28% after extraction [19,20]. Efforts for recombinant expression of hydrophobin HYDPt-1 in *E. coli* produced extremely low yields, just one to two milligrams per liter culture via a traditional IPTG-induced protein production method [21]. Many studies have pursued *Pichia pastoris* as an expression host, as it boasts post-translational modification capabilities superior to *E. coli,* and secretes expressed hydrophobin into the culture media, improving recovery by allowing easier separation of properly folded proteins [22]. Niu et al. achieved a titer of 120 mg/L HFBI in culture supernatant alone through this method, using a *P. pastoris* strain with just one copy of the HFBI gene [23]. The present work seeks to address HFBI production challenges, particularly in post-fermentation recovery and purification, by recombinantly overexpressing HFBI in an engineered *Pichia pastoris* strain containing four copies of the HFBI gene.

Purification challenges arising after hydrophobin production are also well documented in the literature. Linder et al., expressing HFBI in overproducing *T. reesei,* performed separate purification methods for culture supernatant and biomass [20]. Supernatant purification required liquid extraction followed by reverse phase high performance liquid chromatography (RP-HPLC), while HFBI purification from biomass required homogenization, desalting, anion exchange chromatography, and RP-HPLC [20]. Niu et al., expressing in *P. pastoris*, only needed to purify culture supernatant containing secreted HFBI to achieve 92% recovery via ultrafiltration followed by RP-HPLC [23]. Unfortunately, HFBI self-assembly led to fouling of the ultrafiltration membrane, requiring the process to run at a lower pressure of 0.06 MPa and low permeate flux, thus reducing throughput to minimize protein loss [23]. Other researchers have capitalized on this interfacial self-assembly of HFBI and HFBII, employing foam fractionation and foam separation techniques to obtain enriched or purified elution concentrations ranging from 110 mg/L to 360 mg/L [24,25]. These foam separation techniques reduce purities below 70%; shifting to immobilized metal affinity chromatography (IMAC) increases this HFBI purity above 99% while reducing concentration to 300 mg/L, or 30 μM [25]. This lab-scale process reduces a 30 mL culture supernatant into a 1 mL elution fraction, but could be scaled up to process higher volumes of supernatant into larger elution volumes of similar concentration [25]. The present work aims to iteratively improve HFBI production and yields through overproduction of HFBI in *P. pastoris* followed by a novel scalable, automated, and high-throughput single unit operation multimodal chromatography purification method, targeting 5 mL elution concentrations of 40 μM HFBI purified to homogeneity as determined by SDS-PAGE.

## 2. Materials and Methods

### 2.1. Four-Copy HFBI Yeast Transformation

Four-copy N-terminal hexahistidine (His6)-hemaglutinnin (HA)-tagged HFBI pPICZα A plasmids were previously described [26]. *Pichia pastoris* strain GS115 (Invitrogen, Waltham, MA, USA) was made electrocompetent by first inoculating 50 mL YPD (1% yeast extract, 2% peptone, 1% dextrose) in a 250 mL beveled shake flask and growing to saturation overnight at 30 °C and 200 rpm. The entire flask was chilled on ice for 15 min, before pelleting cells in two tubes at 4 °C and 800× *g* for 5 min. Each tube of cells was resuspended in 2 mL YPD containing 200 mM Hepes. To each tube, 400 μL 1 M DTT was slowly added with swirling, then tubes were incubated for 10 min at 30 °C and 100 rpm. Tubes were placed on ice, and 40 mL of sterile, ice-cold water were added to each tube. Cells were pelleted at 4 °C and 800× *g* for 5 min, then washed with another 40 mL of sterile, ice-cold water before pelleting again in the same way. Cells were washed twice with 20 mL sterile, ice-cold 1 M sorbitol at 4 °C and 800× *g* for 5 min each. Finally, cells were resuspended in 300 μL ice-cold sorbitol and divided into 30 μL aliquots for transformation.

As the four-copy HFBI plasmid contains multiple SacI restriction sites, uncut plasmid was transformed into *P. pastoris* stochastically [26]. Still on ice, 10–20 ng plasmids were added to each electrocompetent aliquot and incubated for 5 min. Each mixture was then transferred to a chilled 1 mm electroporation cuvette, which were then dried and pulsed with 1150 V, 25 μF, and 200 Ohms. To quench, 1 mL Pichia Electroporation Recovery Solution (PERS), made of half YPD and half 1 M sorbitol, was quickly added. Electroporated samples were then transferred to tubes and incubated for 3 h at 30 °C and 100 rpm. Finally, samples were spread onto YPD-zeocin plates and incubated at 30 °C to produce colonies.

### 2.2. Strain Selection

Surviving zeocin-resistant colonies were screened for successful stochastic insertion similarly to Sallada et al. [27]. Colonies were inoculated into 400 μL of MGY media (1% yeast extract, 2% peptone, 1.34% YNB, 4 × 10^−5^% biotin, 1% glycerol) in a 2 mL deep 96-well PlateOne polypropylene square well plate (USA Scientific, Ocala, FL, USA). The plate was covered with a sterile Breathe-Easy sealing membrane (Diversified Biotech, Dedham, MA, USA) and grown to saturation overnight at 28 °C and 400 rpm. From each well, 20 μL of saturated culture was extracted to inoculate 400 μL fresh MGY in an identical 96-well plate. This plate was covered and grown to saturation as before. The plate was then centrifuged at 3700× *g* for 10 min to pellet cells. Supernatants were removed and replaced with 400 μL MMY media (1% yeast extract, 2% peptone, 1.34% YNB, 4 × 10^−5^% biotin, 1% methanol) to induce protein expression overnight. The following morning, plates were centrifuged at 3700× *g* for 10 min to pellet cells, and 3 μL supernatant from each well was applied to nitrocellulose membrane (Thermo Scientific, Waltham, MA, USA). This membrane was incubated for 1 h at room temperature in block solution (5% fat-free dry milk in Tris Buffered Saline + 0.1% Tween 20 (TBS-T)) before refrigerating at 4 °C overnight with HRP-conjugated mouse anti-HA tag antibody (Cell Signaling Technology, Danvers, MA, USA) diluted 1:1000 in block solution. The following morning, the membrane was washed 4 times for 5 min each in TBS-T, then treated with Amersham ECL Prime Western blotting Detection Reagents (Cytiva, Marlborough, MA, USA, RPN2232) before imaging on an Amersham 680 Imager (Cytiva).

An HRP-conjugated mouse anti-HA tag antibody Western blot was then performed following manufacturer protocols (Abcam, Cambridge, MA, USA, BioRad, Hercules, CA, USA) on colonies producing high-intensity dots to ensure correct 10 kDa size of expressed protein [28,29]. From each culture corresponding to a high-intensity dot, 18.75 μL supernatant was extracted and added to 5 μL 5× SDS sample buffer dye (0.312 M Tris-HCl, 10% SDS, 50% glycerol, 0.05% bromophenol blue) and 1.25 μL β-mercaptoethanol as a denaturing agent. These 25 μL solutions were boiled at 100 °C for 10 min, then 10 μL of each were loaded into individual lanes of an SDS-PAGE gel and run at 110 V until complete resolution. A piece of nitrocellulose membrane was cut to the size of the completed gel, soaked in SDS-free transfer buffer (4.5 g tris base, 21.6 g, 300 mL methanol, 1200 mL di H_2_O), and placed onto the gel. The gel and paper were then sandwiched between filter paper, sponges, and a gel cassette. The complete cassette was placed into the transfer tank with chilled transfer buffer and run at 100 V for 30 min. The transferred nitrocellulose membrane was then blocked and resolved identically as above.

### 2.3. Chaperone Yeast Transformation

The highest-expression colony was cultured and made electrocompetent as previously described for direct insertion of an additional copy of the chaperone Ero1 gene, as performed by Sallada et al. [26]. A pPIC9 plasmid containing the Ero1gene was previously described [26]. This plasmid was linearized with BspEI (NEB) for direct insertion into the *HIS4* locus, placing the gene under control of the AOXI promoter alongside the HFBI copies. To a tube of 30 μL electrocompetent cells, 5 μg linearized Ero1 pPIC9 vector was added and incubated on ice for 5 min. The mixture was then transferred to a chilled 1 mm electroporation cuvette, which was then dried and pulsed with 1150 V, 25 μF, and 200 Ohms. To quench, 1 mL PERS was quickly added as before. The electroporated samples were then transferred to tubes and incubated for 3 h at 30 °C and 100 rpm. Finally, samples were spread onto minimal dextrose plates without histidine (0.34% YNB without ammonium sulfate or amino acids, 1% ammonium sulfate, 4 × 10^−5^% biotin, 2% dextrose, 2% agar) to select for histidine biosynthesis complementation and incubated until colonies appeared.

### 2.4. Induction Optimization

HFBI production was induced over a five-day shake flask regiment. A single four-copy HFBI plus Ero1 *P. pastoris* colony was picked and inoculated into 100 mL of MGY (1% yeast extract, 2% peptone, 1.34% YNB, 4 × 10^−5^% biotin, 1% glycerol) in a 500 mL baffled flask, then incubated 24 h at 30 °C and 200 rpm. Culture media was then split into two 50 mL conical tubes and centrifuged at 1500× *g* for 5 min. Each pellet was resuspended in 100 mL of MMY (1% yeast extract, 2% peptone, 1.34% YNB, 4 × 10^−5^% biotin, 1% methanol) in a 1000 mL baffled flask, then incubated at 30 °C and 200 rpm for 96 h. At each 24 h mark, 500 μL methanol was added to continue protein expression.

Optimization runs were first performed altering media and feed compositions. Less complex medias YPG (1% yeast extract, 2% peptone, 1% glycerol) and YPM (1% yeast extract, 2% peptone, 1% methanol) were substituted in for MGY and MMY, respectively. Production runs were also performed doubling the daily methanol feed from 500 μL to 1000 μL. Daily and overall induction performance was gauged via wet weight (measured by mass of 1 mL culture pellet), cell density (measured by optical density at 600 nm (OD_600_)), and protein production measured by Bradford assay following the manufacturer protocol (BioRad, Hercules, CA, USA) [30].

### 2.5. Purification Optimization

#### 2.5.1. Harvesting

Cells did not need to be lysed at the end of induction, as *P. pastoris* secretes recombinant HFBI. Culture media was divided into 50 mL conical tubes and centrifuged at 10,000× *g* for 10 min. Supernatant was collected while cell pellets were discarded. After all purification methods, eluted HFBI was dialyzed into pH 5 sodium acetate buffer.

#### 2.5.2. PTFE Wettability Test

This method is adapted from a similar technique by Riccobelli et al. [31]. A half-inch wide strip of hydrophobic polytetrafluoroethylene (PTFE) (Oatey, Cleveland, OH, USA, 31430L) was cut and taped onto a flat surface, pulled flat but not too tight to create a uniform surface. A 20 μL drop of diH_2_O was pipetted onto one end of the tape as a negative control, and 20 μL drops of each test sample were pipetted down the tape in a line from the water drop. Sample labels were placed nearby and not written on the strip to avoid compromising the surface. Amphiphilic behavior reverses the hydrophobicity of PTFE, causing any drops from samples containing surfactants to spread out over a larger surface area than the negative control.

#### 2.5.3. Immobilized Metal Affinity Chromatography (IMAC)

IMAC purification was performed similarly to Mann et al. and Sallada et al. [27,32]. For each 44 mL fraction of culture supernatant, 5 mL 10× IMAC buffer (58.9 g NaCl, 44.4 g Tris HCl, and 26.6 g Tris base per liter buffer), and 1 mL 500 μM imidazole were added. This pH and salt adjusted supernatant was then centrifuged at 10,000× *g* for 10 min, then filtered through 0.2 μm filters (Sartorius, Göttingen, Germany, S6534 FMQ). The IMAC column was first packed with 2 mL HisPur Ni-NTA resin (Thermo Scientific, Waltham, MA, USA, 88221) and washed with 10 column volumes (CV) diH_2_O. The column was then equilibrated with 3 CV 10 mM imidazole, then all the filtered culture supernatant was poured onto the column for affinity binding between the resin and N-terminal His tag. After a 10 CV 25 mM imidazole wash, HFBI was eluted with 3 CV 500 mM imidazole. HFBI presence in the elution was checked via Western blot.

#### 2.5.4. Size Exclusion Chromatography

PD-10 Sephadex G-25 desalting columns (Cytiva, Marlborough, MA, USA, 17085101) were first used to purify HFBI from culture supernatant via the following provided spin protocol [33]. The cap, storage solution, filter, and end seal were all removed from the column. After stabilizing the column on a clamp, 25 mL of water were poured over the column to equilibrate. When liquid flow from the column stopped, the column was placed in a 50 mL conical tube with the supplied adapter and centrifuged at 1000× *g* for 2 min. The column was moved to a fresh 50 mL conical tube, 2.5 mL culture supernatant were poured onto the column, and HFBI was eluted by centrifuging at 1000× *g* for 2 min.

The following provided gravity protocol was also investigated. The cap, storage solution, and end seal were once again removed, while the filter was left in place. After stabilizing the column on a clamp, 25 mL of water were poured over the column to equilibrate. When liquid flow from the column stopped, 2.5 mL culture supernatant were poured onto the column. To elute, 20 mL of water was slowly poured onto the column, and 1 mL fractions were collected in 1.5 mL tubes.

#### 2.5.5. Multi-Modal Cation Exchange Chromatography

**Figure 2:**
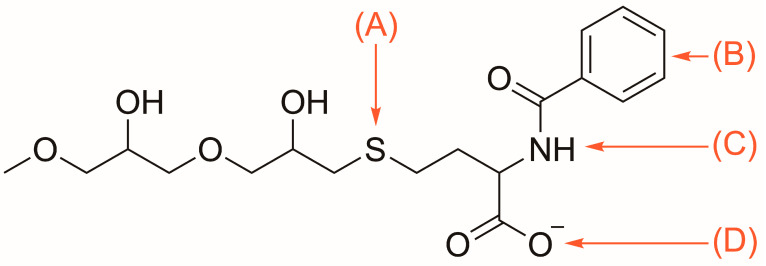
Capto MMC ImpRes ligand featuring: (A) thiophilic; (B) hydrophobic; (C) hydrogen bond; and (D) electrostatic affinity regions [34].

Capto MMC ImpRes (Cytiva, Marlborough, MA, USA), a tetra-modal cation exchange resin featuring electrostatic, hydrophobic, thiophilic, and hydrogen bond affinity regions on each ligand, was selected for its four-mode affinity to HFBI (Figure 2) [34]. As the isoelectric point of recombinant HFBI is 5.9, HCl was added to the culture supernatant to impart a positive charge at pH 4.5 [35]. After pH adjustment, culture supernatant was centrifuged at 10,000× *g* for 10 min and filtered through 0.2 μm filters (Sartorius, Göttingen, Germany, S6534 FMQ) before loading into the ÄKTA Pure.

This purification was performed as follows according to the column manufacturer suggested protocol [36]. The column was equilibrated with 10 CV pH 4.5 25 mM sodium acetate before loading HFBI supernatant onto the column. After a 5 CV wash of pH 4.5 25 mM sodium acetate, HFBI was eluted in fractions by an increasing salt/pH gradient as the feed composition gradually transitioned over 20 CV into 50 mM sodium phosphate with 1 M ammonium chloride at pH 7.2. Beyond manufacturer instructions, purifications were also performed eluting with 1 M and 0.1 M sodium hydroxide instead of 50 mM sodium phosphate with 1 M ammonium chloride.

Multiple purification runs were performed varying supernatant load volumes from 15 mL to 100 mL. Two sizes of Capto MMC ImpRes column were tested, both the 1 mL HiTrap (Cytiva, 29401108) and 4.7 mL HiScreen (Cytiva, Marlborough, MA, USA, 17371620). HiTrap elution fractions were 2 mL each, while HiScreen elution fractions were 5 mL each. Flow rates and maximum pressures for each of these column models were set to manufacturer suggestions.

## 3. Results

### 3.1. Optimizing Induction

**Figure 3:**
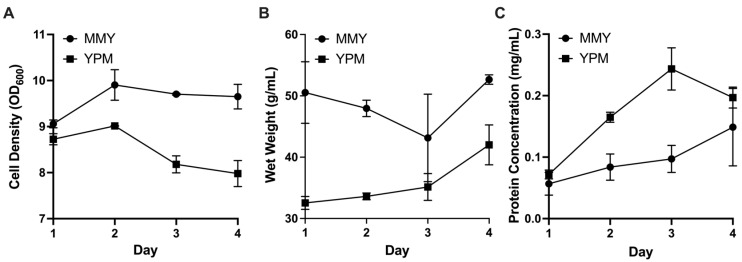
*P. pastoris* induction optimization parameters over the course of 4 days to compare complex media (MMY) with low complexity media (YPM): (**A**) Cell density measured by OD_600_; (**B**) culture wet weight measured in g/mL; and (**C**) total protein concentration in mg/mL measured by Bradford assay (error bars, representing ±1 standard deviation, are in some instances smaller than data point markers themselves).

After performing identical production runs with MGY/MMY (complex) and YPG/YPM (lower complexity) medias, each in duplicate, there was no measured benefit of complex media when comparing final protein concentrations. Although complex media slightly outperformed lower complexity media with respect to cell density and wet weight, YPM produced higher protein yields at the end of four induction days (Figure 3). Doubling the methanol dosage did not have a detectible benefit for final protein production, measured by A280.

### 3.2. Optimizing Purification

All HFBI fractions were dialyzed into pH 5 sodium acetate buffer before quantification by Nanodrop A280. This method relies on the tryptophan residue within the HFBI sequence, and calculates concentration from absorbance utilizing the extinction coefficient of 11,960 L/mol-cm.

**Figure 4:**
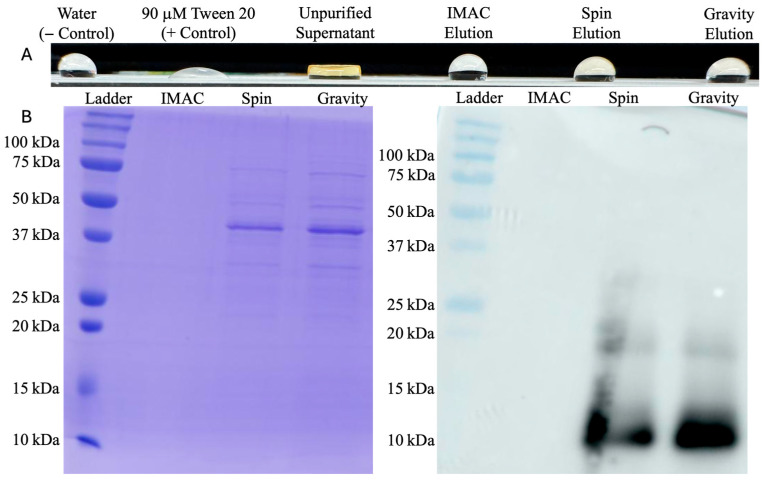
HFBI purification assessment: (**A**) contact-angle testing on PTFE showing stronger surfactant activity in unpurified supernatant than purified products; and (**B**) twin SDS-PAGE and HRP conjugated mouse anti-HA tag antibody Western blot showing purity and presence of HFBI for each preliminary purification method. HFBI concentrations of spin and gravity protocol elutions are below SDS-PAGE limit of detection; the 10 kDa bands are only detectable in the more sensitive immunoblots.

From 200 mL of culture supernatant, IMAC purification averaged 10 μM HFBI in a 6 mL elution. This 200 mL production and purification protocol was performed 8 separate times, with all final product concentrations between 8 and 12 μM. Performing a wettability test on PTFE after each run, unfiltered and unpurified HFBI supernatant from this overexpressing strain demonstrated stronger surfactant activity than purified product (Figure 4). In tandem with the Western blot below (Figure 4) showing HFBI presence in other purification methods from the same supernatant, this indicated a purification problem rather than a production problem. Both centrifugal spin and gravity drip modes of size-exclusion chromatography were then investigated as alternatives.

PD-10 desalting columns were first used with the manufacturer spin protocol (Cytiva) [33]. Loading two duplicate desalting columns with 2.5 mL of culture supernatant and centrifuging produced 2.5 mL of impure protein elution averaging 180 μM. Although this method produced much higher overall protein recovery than IMAC, the SDS-PAGE above (Figure 4) indicated many culture media components and yeast host cell proteins were centrifuged into the elution as well.

The gravity purification protocol using PD-10 desalting columns was then pursued as an alternative. Two duplicate columns were loaded with 2.5 mL of culture supernatant once again but eluted over a larger volume of 20 mL to separate HFBI from contaminants. With a size of 10 kDa, HFBI is one of the first proteins to elute from the column and was found to be in the second 1 mL fraction of each column with concentrations averaging 60 μM. SDS-PAGE above (Figure 4) indicates a similarly impure protein distribution as the elution obtained from the spin protocol. Based on the highly impure elutions produced from both spin and gravity protocols, size exclusion chromatography was determined to be an ineffective method of HFBI purification, creating the need to pursue a higher fidelity purification method.

Next, an ÄKTA Pure system was used with Capto MMC ImpRes columns for purification. Before loading onto the ÄKTA, culture supernatant pH was lowered beneath the HFBI isoelectric point of 5.9 to impart a positive charge on the protein for electrostatic interactions with Capto MMC ImpRes [35]. Adjusting the pH to 4.5 caused some supernatant impurities to precipitate, which were removed by centrifugation and filtration before ÄKTA purification.

**Figure 5:**
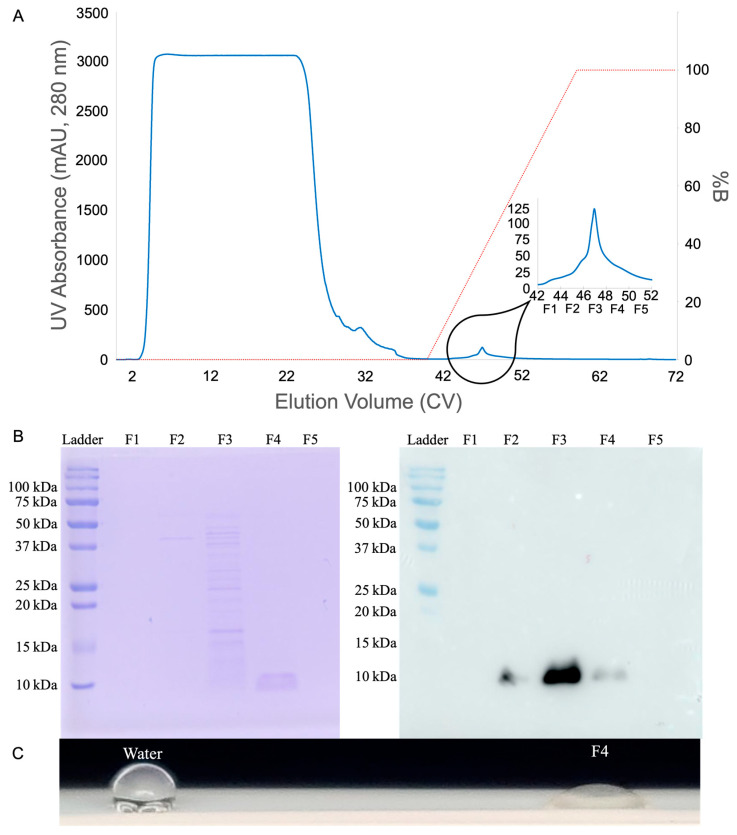
HFBI purification quality control: (**A**) chromatogram marking five 2 mL elution fractions, labeled F1-F5, spanning the width of the elution peak; (**B**) twin SDS-PAGE and HRP conjugated mouse anti-HA tag antibody Western blot showing pure 10 kDa band in elution fraction F4 and HFBI presence in fractions 2–4; and (**C**) contact-angle testing on PTFE shows strong surfactant function.

Starting with a 1 mL Capto MMC ImpRes HiTrap column, each loading volume was tested in duplicate. The major elution peak was captured, and after dialysis the Nanodrop A280 was used to measure concentrations of 5.4 to 7.5 μM HFBI in a 2 mL elution from 20 mL of loaded supernatant. Loading 40 mL HFBI produced 14 to 18 μM HFBI in a 2 mL elution, loading 50 mL HFBI produced 22 to 24 μM HFBI in a 2 mL elution, but loading 100 mL only produced 20 to 26 μM HFBI in a 2 mL elution. For each of these, presence of product in the elution was confirmed by SDS-PAGE, Western blot, and qualitative PTFE wetting experiments (Figure 5).

To scale up production, the 1 mL column was replaced by a 4.7 mL Capto MMC ImpRes HiScreen column. Loading 100 mL HFBI onto this column produced an average of 15 μM HFBI in a 5 mL elution across two purification runs. This reduction in yield was accompanied by high pre-column pressure throughout purification. To address these problems, 1 M ammonium chloride was replaced with 0.1 M sodium hydroxide in the elution buffer to more effectively elute HFBI. Performing three runs loading 15 mL HFBI onto this column with modified elution buffer yielded 14 to 26 μM HFBI in 5 mL fractions. Performing three runs loading 25 mL HFBI onto this column with modified elution buffer yielded 28 to 32 μM HFBI in 5 mL fractions. Increasing load volumes to 50 mL increased yield concentration into the range of 45 to 53 μM HFBI in 5 mL fractions across three runs. These purification results are summarized in Table 1.

## 4. Discussion

Neither increasing methanol feed nor including YNB and biotin in the fermentation media led to improved protein induction. These additional reagents would also add considerable cost to HFBI production, and thus are not useful for eventual larger-scale production. For this reason, future production runs were performed with YPG and YPM media instead of complex MGY and MMY. Cell density was first maximized in carbon-rich YPG media for 24 h, then induced in YPM media with 500 μL feeds up to 0.5% methanol daily for four days.

IMAC was selected as the initial HFBI purification method due to previous success described in the literature [25,27]. This method was pursued well before completing the strain optimization described in Section 2.2 and Section 2.3 with a low expression single-copy HFBI strain. Yields remained low, averaging 10 μM, which is a third of the yield achieved via similar IMAC purification by Lohrasbi-Nejad et al. [25]. Moreover, protein bands were not observed on SDS-PAGE, even after completing strain optimization, necessitating the use of other purification methods.

PD-10 desalting columns, used either with spin or gravity protocols, created a different problem: Western blots showed HFBI content, but SDS-PAGE indicated very low concentration and purity, as shown in Figure 4. These lingering host cell proteins inflate final concentration reads to 60 or 180 μM for gravity and spin protocols, respectively, and can interfere with HFBI function. In addition, this form of size exclusion chromatography drastically reduced purification throughput, only processing 2.5 mL culture supernatant each run compared to the 200 mL processed by an IMAC run.

An ÄKTA Pure system was pursued to further automate purification and optimize both throughput and purity. This fully programmable system could run multiple load/elute purifications in sequence when loaded with HFBI supernatant sample, equilibration and elution buffers, and elution collection tubes. Built-in UV sensors mark elution peaks with their coordinates in the fraction collector, removing the fraction screening step characteristic of the PD-10 gravity protocol. Both 1 mL HiTrap and 4.7 mL HiScreen columns were tested to evaluate early-stage scalability of HFBI production utilizing Capto MMC ImpRes.

The tetra-modal cation exchanger Capto MMC ImpRes features four different forms of affinity towards HFBI: Thiophilic, hydrophobic, electrostatic, and hydrogen bond [34]. The thiophilic affinity region is attracted to sulfur, which appears eight times in HFBI, once on each cysteine residue. The hydrophobic benzene ring is attracted to the hydrophobic patch of HFBI. The deprotonated carboxyl group is conditionally electrostatically attracted to HFBI when the protein is below the isoelectric point and carrying a positive charge. Lastly, hydrogen bonds provide affinity for multiple other residues found in the HFBI sequence. When culture supernatant was titrated to pH 4.5, well under the isoelectric point of 5.9, the positively charged HFBI could leverage all four modes of affinity to the resin. Most other host cell and media proteins did not have as robust affinity towards the resin, and flowed through or were washed off prior to HFBI elution as shown by the chromatogram and SDS-PAGE screening of elution fractions F1-F5 in Figure 5.

The potential of Capto MMC ImpRes purification was immediately realized through the results shown in Figure 5. Although initially of low concentration, the purity and throughput persisted throughout the early-stage scale-up described above. Although HFBI is present in fractions F2-F4, as shown by the Western blot in Figure 5B, the parallel SDS-PAGE shows only fraction F4 contains HFBI purified to homogeneity. This indicates HFBI begins to elute alongside contaminants earlier in the salt/pH gradient, but continues eluting later in the gradient after contaminants have been removed.

The 1 mL HiTrap column reached an operational limit when loaded with 50 mL culture supernatant; pushing this load volume higher did not further increase elution concentration. Although Capto MMC ImpRes has a very high protein binding capacity, the strong amphiphilic behavior of HFBI may have caused aggregation and premature saturation.

Scaling up to the 4.7 mL HiScreen column did not immediately improve yields with the same 100 mL loading. The column was eluted as normal with 50 mM sodium phosphate with 1 M ammonium chloride at pH 7.2, then washed with 1 M sodium hydroxide. The UV sensor detected a strong signal during this wash, indicating large amounts of protein remaining on the column even after elution. Replacing the elution buffer with sodium hydroxide solved the problem of concentration. Initial runs utilizing 1 M sodium hydroxide eluted everything off the column in one 5 mL fraction, even on the same elution gradient as before. Reducing this buffer concentration to 0.1 M creates the ideal separation of fractions for pure HFBI elution. As shown in Table 1, HiScreen elution concentrations surpassed those of HiTrap, even when loading a fraction of the volume. Purification returns began diminishing when scaling up from 25 mL to 50 mL loadings, so higher volume loads (i.e., 100 mL) were not pursued.

When eluting with 0.1 M sodium hydroxide, initial HFBI fractions had very high protein concentrations. These were also eluted in a harsh high-pH buffer, so were dialyzed into pH 5 sodium acetate buffer to avoid degradation. Protein concentration was reduced during dialysis; snakeskin tubing with molecular weight cutoff of 3500 retained HFBI while allowing smaller impurities to exit the solution. Similarly to proteins precipitating after titrating culture supernatant to pH 4.5 before chromatography, this step affords additional purification after chromatography. On the contrary, some HFBI may be lost by binding to the dialysis tubing [25]. Only post-dialysis concentrations are recorded in Table 1.

By multiplying Table 1 elution fraction volumes by their concentrations and dividing by load volume, the highest theoretical culture yield was found to be 86.6 mg/L from the 15 mL culture load on the 4.7 mL HiScreen column. This theoretical titer is higher than some previously published hydrophobin productions, specifically 48.6 mg/L HFBII produced in *T. reesei* and 1–2 mg/L HYDPt-1 produced in *E. coli* [18,21]. Neither of these protocols engineered the host organism for overproduction with multiple copies of the hydrophobin gene; this change alone may explain the increased yield of the present work. Additionally, simple shake flasks were used here instead of specially designed biofilm reactors seen in the literature, further strengthening the value of this presented method once implemented in an optimized bioreactor workflow [18]. Although not as high as some other HFBI production yields, such as the published 600–700 mg/L from overproducing *T. reesei*, none of the produced HFBI was trapped in mycelia [19,20]. The consequences of this arise during purification. When overproducing HFBI in *T. reesei*, so much throughput is lost by the necessity of separately preparing and purifying the supernatant with two-unit operations and the biomass with four-unit operations [20]. Each of these steps reduces recovery, ultimately leading to just 14 mg/L HFBI produced from the supernatant [20]. Using the overproducing *P. pastoris* strain and multimodal chromatography methodology presented in this work, these six-unit operations are reduced to one, but still with the required steps of sample preparation. This single unit operation simplicity reduces opportunities for protein losses at each step, simplifies scale-up to fewer machines, and reduces time expended to complete each purification.

The multimodal chromatography method presented here also addresses volumetric throughput challenges found in the literature. Niu et al. achieved an HFBI titer of 120 mg/L, higher than the 86.6 mg/L of the present work, from their *P. pastoris* strain, but were unable to run the purification ultrafiltration at maximum pressure due to membrane fouling [23]. During ÄKTA purifications performed for this work, column volumetric flow rates were always run at the manufacturer suggested maximum. Purified 1 mL HFBI elutions with concentrations of 30 μM produced via foam fractionation and purified via IMAC by Lohrasbi-Nejad et al. were surpassed in volume and concentration by the 5 mL HFBI elutions with concentrations of 48 μM produced in the present work [25]. Higher volumetric yields, automatic operation, and scalability of this single unit operation purification process show industrial promise [24]. Additionally, the emergent customizability of this method can be seen here. Loading only 15 mL onto the column yields the highest theoretical culture yield, but with a lower concentration of 26 μM. If needed for a specific application, overall recovery can be sacrificed to yield a higher concentration elution, such as 48 μM when loading 50 mL.

Looking toward the future, the HFBI production protocol presented here could be further improved by implementing bioreactor fermentation in leu of shake flask fermentation. Bioreactors provide additional fermentation controls leading to increases in biomass production and overall product yield [37,38,39,40,41]. Bioreactors may also automatically introduce antifoam to mitigate the inevitable foaming of biosurfactant production alongside increased aeration.

As seen in Figure 5B, some HFBI product is eluted earlier in the salt/pH gradient alongside contaminants. These losses could be reduced in the future by lowering elution fraction volumes to better separate contaminants from products, then pooling pure elution fractions. Additionally, to maximize purified yield from fermentation, impure fractions such as F2 and F3 could be collected and recycled in a later purification. Capto MMC ImpRes column size could be increased to allow even higher throughput of this purification. Additionally, protein aggregation is one of many possible variables to explore and optimize in the future.

This protocol will also be used in ongoing and future studies for production and purification of HFBI mutants screened to exhibit enhanced performance in lignocellulosic biomass conversion, and should also provide value in streamlining production using future strain engineering approaches to further boost titers and overcome limitations of lost material in biomass during extraction.

## 5. Conclusions

Through an iterative approach, optimal HFBI production was determined as a 5-day production regiment in simple culture media, followed by multi-modal cation exchange chromatography purification. Capto MMC ImpRes multi-modal cation exchange via an ÄKTA Pure system yielded the highest throughput and protein purity. High-volume HFBI yields of 86.6 mg/L were produced and purified to 48 μM from this single unit operation, improving upon methodologies found in the literature [18,21]. The purification protocol presented was pursued for scalability; multiple batches can be induced in parallel, and batch and column size can be increased, even to the production scale of industrial bioreactors.

## Figures and Tables

**Table 1:** Capto MMC ImpRes performance overview.

Column	Load Volume [mL]	Elution Fraction Volume [mL]	Elution Concentration [μM]
1 mL HiTrap NH_4_Cl elution	20	2	5.4–7.5
	40	2	14–18
	50	2	22–24
	100	2	20–26
4.7 mL HiScreen NaOH elution	15	5	14–26
	25	5	28–32
	50	5	45–48

## Data Availability

The original contributions presented in this study are included in the article. Further inquiries can be directed to the corresponding author.

## Abbreviations

The following abbreviations are used in this manuscript:

IPTG	Isopropyl β-D-1-thiogalactopyranoside
His6	Hexahistidine
HA	Hemaglutinnin
YNB	Yeast Nitrogen Base
PERS	Pichia Electroporation Recovery Solution
HRP	Horseradish peroxidase
TBS-T	Tris-buffered saline with Tween 20
SDS	Sodium dodecyl sulfate
NEB	New England Biolabs
AOXI	Alcohol Oxidase 1
PTFE	Polytetrafluoroethylene
IMAC	Immobilized Metal Affinity Chromatography
CV	Column Volume
MMC	Multimodal Chromatography
REU	Research Experience(s) for Undergraduates
